# UHPLC-ESI-MS/MS Quantitative Analyses of Multicomponent Hu Gan Tablets

**DOI:** 10.3390/molecules24234241

**Published:** 2019-11-21

**Authors:** Jun Liang, Xin-Dong Guo, Fang Han

**Affiliations:** Key Laboratory of Chinese Materia Medica Ministry of Education, Heilongjiang University of Chinese Medicine, Harbin 150040, China; guoxindong007@163.com (X.-D.G.); HanFang1109@163.com (F.H.)

**Keywords:** UHPLC-ESI-MS/MS, simultaneous determination, Hu Gan tablets, quality guarantee

## Abstract

Nowadays, the analysis of the multi-components in Chinese patent medicine prescriptions is being paid more attention. Therefore, in this study for the first time, a simple, rapid ultrahigh performance liquid chromatography–electrospray ionization tandem mass spectrometry (UHPLC-ESI-MS/MS) method was established for simultaneous determination of 18 active compounds in a Chinese patent medicine of Hu Gan tablets (HGT) from different pharmaceutical factories in China. This task has met great emerging challenges from not only structural complexities and similarities but also co-occurrence of water-soluble and fat-soluble components in HGT. UPLC-ESI-MS/MS was put forward to solve the problems. It was operated in both positive and negative mode using multiple reaction monitoring (MRM). The mobile phase was 0.1% formic acid in water (A) −0.1% formic acid in acetonitrile (B) with linear gradient elution at a flow rate of 0.2 mL/min, run for a total of 12.0 min. The optimized method used provided short analysis time and good linearity (*R*^2^ > 0.99), and intra- and inter-day precision (relative standard deviation (RSD) < 4.00%) with good accuracy (94.89–110.03%) and recovery (70.00–126.09%). The results indicate the method could be practically used for quality guarantee of HGT and might also be useful for further studies.

## 1. Introduction

Hu Gan tablets (HGT) have been traditionally applied to treat hepatic fibrosis diseases. This Chinese patent medicine includes *Bupleuri radix* (Chaihu), gapillary wormwood (Yinchen), *Schisandrae chinensis fructus* (Wuweizi), *Isatidis radix* (Banlangen), *Suis fellis pulvis* (Zhudanfen), and *Phaseolus radiatus* (Lüdou) [1,2]. Because HGT possesses huge consumer market potential for the treatment of fatty liver disease, alcoholic liver disease, drug-induced liver injury, chronic hepatitis, and early cirrhosis [3,4,5,6,7,8], the quality guarantee is of vital importance. It is well-known that the main bioactive components of HGT are lignans, organic acids, flavonoids, alkaloids, coumarins, saponins, and bile acids [5,6,7]. These chemical ingredients have been proven to be responsible for the various biological activities of this Chinese formula [8].

Chaihu contains triterpene saponins such as saikosaponins A, D, E, F, and H [9]. Saikosaponins are commonly used to cure liver damage [10], and anti-inflammatory [11]. Yinchen has been shown to possess liver protection, blood pressure lowering, antipyretic, anti-inflammatory, antibacterial, anti-microbial, and antitumor activity, and is one of the oldest medicinal herbs [12]. It has been used to treat acute icteric infectious hepatitis, hyperlipemia, and oral ulcers. Banlangen is one of the most commonly used plants in TCMs for anti-viral, anti-cancer, anti-bacterial and immune enhancement [13]. Some researchers have also found that Wuweizi has pharmacological effects which reduce transaminase and anti-hepatic injury [14]. It has been reported that dibenzocyclooctadiene lignans are responsible for the major biological function of Wuweizi. Zhudanfen has been used extensively for the treatment of dysentery, jaundice, diarrhea, acute pharyngitis, asthma, and whooping cough [15]. Flavonoids and phenolic acids of Lüdou can treat cancer, cardiovascular diseases, ageing, and diabetes [16].

Many methods have been reported in the literature for measurement of saikosaponins and dibenzocyclooctadiene lignans. These methods include high performance liquid chromatography with diode array detection (HPLC-DAD) [17], HPLC with mass spectrometry (HPLC-MS) [18], HPLC with evaporative light scattering detection (HPLC-ELSD) [19], and RP-HPLC [20]. Although these methods have also been applied for quantification of saikosaponins from the HGT [17], a simultaneous analysis of flavonoids, lignans, and phenylpropanoids in this prescription through ultrahigh performance liquid chromatography–electrospray ionization tandem mass spectrometry (UHPLC-ESI-MS/MS) based on multiple reaction monitoring (MRM) for speedy quality evaluations is still missing.

**Figure 1:**
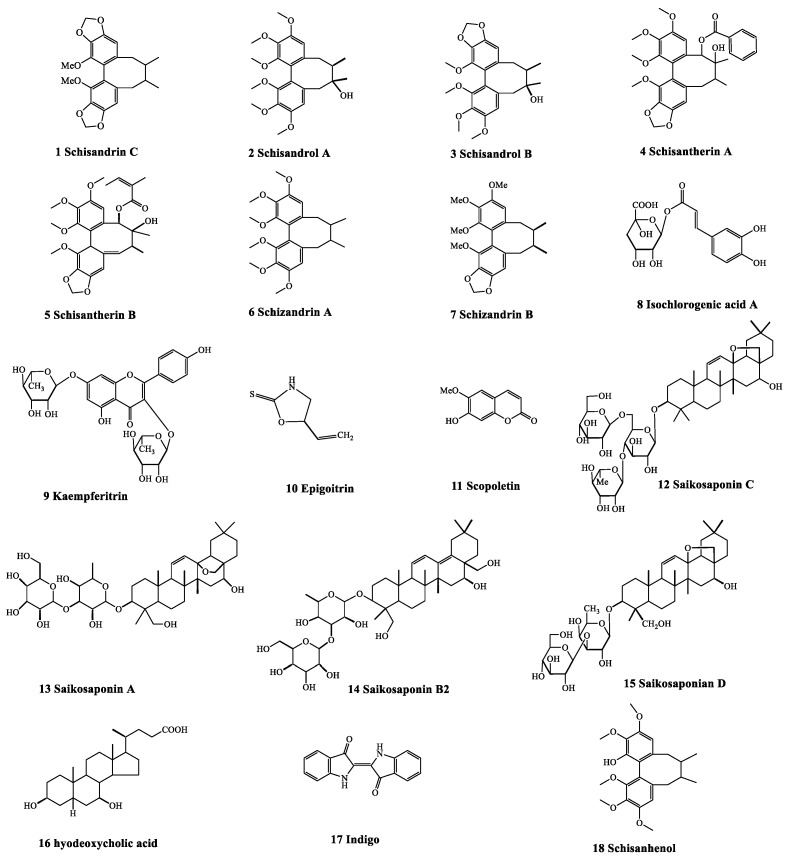
The structures of 18 reference standards in Hu Gan tablets.

UHPLC is based on available reverse phase chromatographic media with a 1.7 um particle size, together with a liquid system that can operate such columns at much higher pressures. In comparison with traditional HPLC, UHPLC–MS offers many advantages, including higher separation efficiency, shorter analysis time, and less solvent consumption. Furthermore, it offers the possibility to obtain a more comprehensive chemical profile and quantization by utilizing different ion modes and through having high sensitivity [21,22,23]. In this study, we established a rapid UHPLC-ESI-MS/MS method to determine 18 compounds in HGT (Figure 1) with a MRM mode. UHPLC-ESI-MS/MS also has the advantages of enabling analysis of low-content compounds and saving analysis time.

## 2. Results and Discussion

### 2.1. Optimization of UHPLC-ESI-MS/MS Conditions

**Figure 2:**
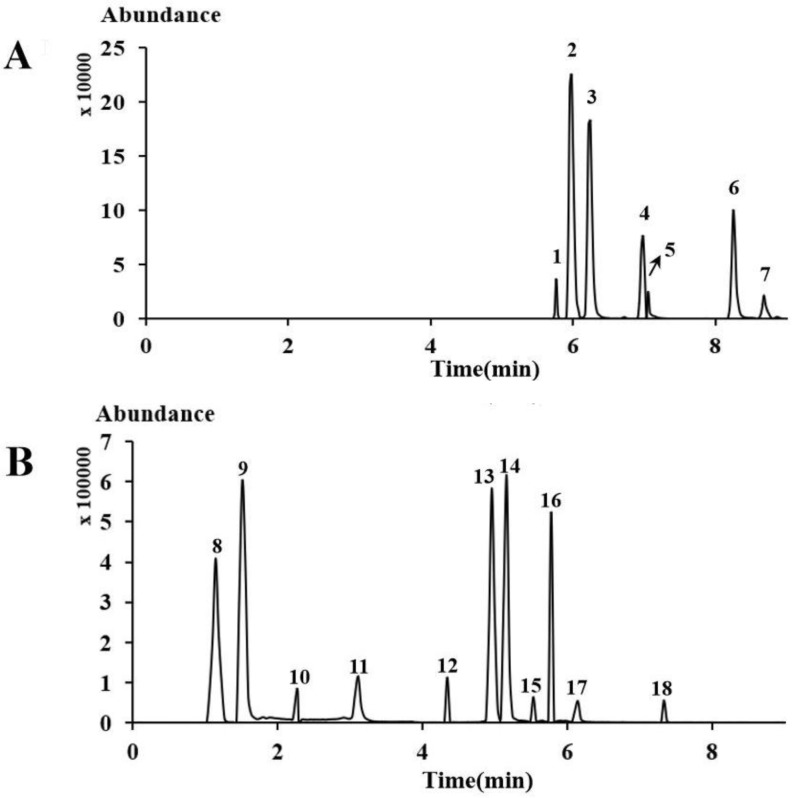
Multiple reaction monitoring (MRM) total ion chromatograms of 18 reference standards in (**A**) positive mode and (**B**) negative mode.

Three reversed-phase chromatographic columns, including a HSS T3 (1.8 um, 2.1 mm × 100 mm), BEH C_18_ (1.7 um, 2.1 mm × 100 mm), and Cortecs C_18_ (1.6 um, 2.1 mm × 100 mm), were tested with the same sample solution. The results showed that the HSS T3 column displayed acceptable separation capacity. The ACN/H_2_O system provided the best performance through the optimization of different mobile phases (MeOH/H_2_O, ACN/H_2_O, and ACN/MeOH/H_2_O). Several different modifiers were investigated (no modifier, formic acid, and ammonium formate), and the results showed that formic acid provided the best peak shape. Additional UHPLC conditions were optimized by varying column temperatures (25, 30, 35, and 40 °C), and flow rates (0.2, 0.3, and 0.40 mL/min). The optimized UHPLC conditions provided the highest selectivity and resolution. These were: HSS T3 column at 35 °C, 0.1% formic acid in water (A)–0.1% formic acid in acetonitrile (B) mobile phase gradient at a flow rate of 0.20 mL/min. A total of 18 compounds tested were submitted in the presence of the [M − H]^−^, [M + H_2_O − H]^−^ in negative mode as well as [M + H]^+^ and [M + Na]^+^ in positive mode. Thus, a 4000 QTRAP equipped with an ESI interface in positive and negative modes was used for detection. As shown in Figure 2, reference standards **1**–**18** showed good peak shapes and excellent resolutions (R > 1.46). The main MS parameters, including declustering potential (DP) and collision energy (CE), were acquired and are summarized in Table 1.

### 2.2. Comparisons with Other Analytical Methods

Previous reports on analysis of HGT always adopt HPLC, however, only several limited components have been quantified, such as schizandrin, which is often used as the quantitative component of Wuweizi [24], and chlorogenic acid, which is often used as the quantitative component of Yinchen [25]. The mobile phase was methanol–water with linear gradient elution at a flow rate of 1.0 mL/min, run for a total of 85.0 min for quantitative determination of 3 lignans, namely schisandrin, deoxyschisandrin, and schizandrin B. The RP-HPLC mobile phase was acetonitrile–water with 60.0 min gradient elution program at 1.0 mL/min for quantitative determination of 4 lignans (schizandrol A, schisantherin A, deoxyschisandrin, and schisandrin) in HGT [20]. To develop a RP-HPLC method for the determination of chlorogenic acid in HGT, a mobile phase of acetonitrile–0.4% phosphoric acid solution (*v*:*v* = 13:87) was used at 0.8 mL/min for 30.0 min [26]. It can be seen that at least 24 mL organic solvent was consumed in one cycle of HPLC injection. Compared with other analytical methods, the UHPLC-ESI-MS/MS method possessed quite a wide linear range and good sensitivity. For instance, the LOQs were 9.77 × 10^−3^ μg/mL for schisandrol A, schisandrol B and schizandrin A (**2, 3** and **6**), as well as 0.02 μg/mL for schizandrin B (**7**), 18, 18, 15, and 7 times lower than those from GC-MS detection method, respectively [27].

As shown in Figure 1, a new UHPLC-ESI-MS/MS method was readily established in this study for simultaneous determination of 18 compounds in HGT (schisandrin C (**1**), schisandrol A (**2**), schisandrol B (**3**), schisantherin A (**4**), schisantherin B (**5**), schizandrin A (**6**), schizandrin B (**7**), isochlorogenic acid A (**8**), kaempferitrin (**9**), epigoitrin (**10**), scopoletin (**11**), saikosaponin C (**12**), saikosaponin A (**13**), saikosaponin B_2_ (**14**), saikosaponian D (**15**), hyodeoxycholic acid (**16**), indigo (**17**), and schisanhenol (**18**)) using 0.1% formic acid in water and 0.1% formic acid in acetonitrile as the mobile phase, at a flow rate of 0.2 mL·min^−1^ for 12 min. It is obvious that only 2.4 mL organic solvent was consumed in one cycle of UHPLC-ESI-MS/MS injection. This fact showed that the proposed analytical method was not only environmentally friendly but also a more efficient approach for quantitative analysis. Therefore, ultrahigh performance liquid chromatography–electrospray ionization multiple reaction monitoring tandem mass spectrometry (UHPLC- ESI- MRM-MS/MS) was confirmed to be a simple and rapid method for HGT samples, characterized by being solvent-efficient and environmentally promising.

### 2.3. Method Validation

The results of the calibration curve are summarized in Table 2. Good correlations were observed between the peak area (*y*) and concentrations of tested compounds (*x*, μg/mL) (*R*^2^ ≥ 0.99) within test ranges. The limit of detection (LOD, signal-to-noise ratio (S/N) = 3) and the limit of quantification (LOQ, S/N = 10) values for all standard analyses were in the range of 2.44–625.00 ng/mL and 9.77–1250.00 ng/mL, respectively. There results suggested that this method is sensitive for the quantitative determination of major components in HGT samples. In order to verify the reliability, the same preparation procedure was adopted for analysis of six different samples. The results showed that the RSD values of component contents and retention times of these 18 compounds were all less than 3.0%, which satisfied the quantitative analysis criteria. The intra-day precision for each compound was assessed by measuring a standard mixture solution composed of 18 compounds at low, medium, and high concentrations on one day, while inter-day precisions were evaluated six times a day on three consecutive days. The results indicated that the mean precision and RSD were less than 4.0% for all compounds. In term of stability, most of the compounds’ reproducibility was acceptable, proven by analysis of one sample at different times and different levels on 3 days, with no significant differences. This proved the sample solution was stable at room temperature for at least 3 days. Results from determination of intra-day and inter-day precision (as RSD) are shown in Table S1.

Recovery experiments were performed at three concentration levels (low, medium, and high levels) by adding an appropriate amount of standard solution to the blank samples. Then, the mixed samples were extracted and analyzed with the established method and triplicate experiments were performed at each level. Recoveries were calculated by the formula: recovery (%) = (detected amount − original amount)/spiked amount × 100%. As shown in Table S2, the RSD values were in the range of 0.08–3.00% and recoveries of analyses varied from 70.00% to 126.09%.

### 2.4. Sample Analysis

HGT is a traditional Chinese medicine patent prescription consisting of Chaihu, Yinchen, Wuweizi, Banlangen, Zhudanfen, and Lüdou [28]. According to previous reports, most research has focused on the pharmacological activity and content determination of one or two indicative compounds in single-herb medicines from HGT [2]. Few studies have paid attention to the entire HGT formulation. To date, no reports have been found covering simultaneous determination of multiple types of compounds from multiple herb plants in HGT using a single method. Consequently, it is necessary to develop an effective and reliable method to analyze as many HGT constituents as possible to ensure its safety and efficacy.

The validated method was successfully applied for quantification of 18 active compounds in eight batches of HGT samples. These 18 chemical compounds were unambiguously classified into seven structural types, namely lignans, organic acids, flavonoids, alkaloids, coumarins, saponins, and bile acids. It is well-known that schisandrin C (**1**), schisandrol (**2**), schisandrol B (**3**), schisantherin A (**4**), schisantherin B (**5**), schizandrin A (**6**), schizandrin B (**7**), and schisanhenol (**18**) are from Wuweizi [29], aikosaponin C (**12**), saikosaponin A (**13**), saikosaponin B_2_ (**14**), and saikosaponian D (**15**) are from Chaihu [30], aisochlorogenic acid A (**8**) and scopoletin (**11**) are from Yinchen [31,32], epigoitrin (**10**) and indigo (**17**) are from Banlangen [33,34], and flavonoid glycoside (**9**) and hyodeoxycholic acid (**16**) are from Lüdou [35] and Zhudanfen [36], respectively. These constituents covered major types of compounds from different single-herb medicines. At the same time, this is the first simultaneous determination of these eighteen active compounds in HGT based on the UHPLC-ESI-MRM-MS/MS method. Therefore, this study may provide an effective reference for quality control of HGT for pharmaceutical manufacturers.

**Figure 3:**
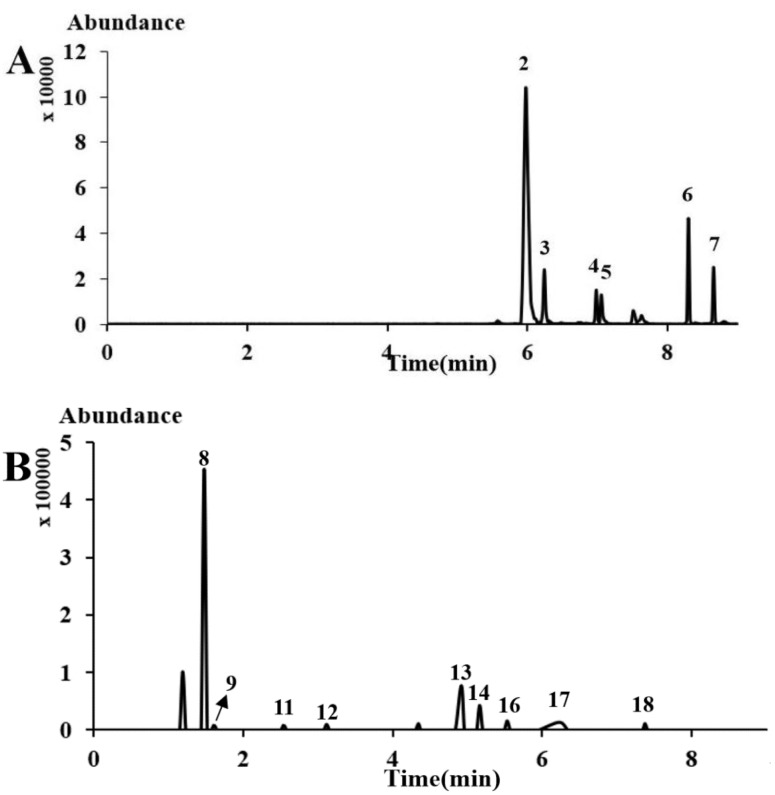
Typical multiple reaction monitoring (MRM) chromatograms of sample **6** in (**A**) positive mode and (**B**) negative mode.

The contents of the investigated compounds, based on their respective calibration curves, are summarized in Table 3. Among these compounds, schisandrol (**2**) was very abundant in 8 batches of HGT samples (Figure 3A). In addition, aisochlorogenic acid A (**8**) was also found to be the most dominant constituent out of all samples tested, at amounts of 67.5–160.7 μg/g (Figure 3B). On the contrary, three compounds, namely schisandrin C (**1**), epigoitrin (**10**), and saikosaponian D (**15**), were not detected at all. The three reference standards that were not detected may be explained by the specific processing method used for HGT.

**Figure 4:**
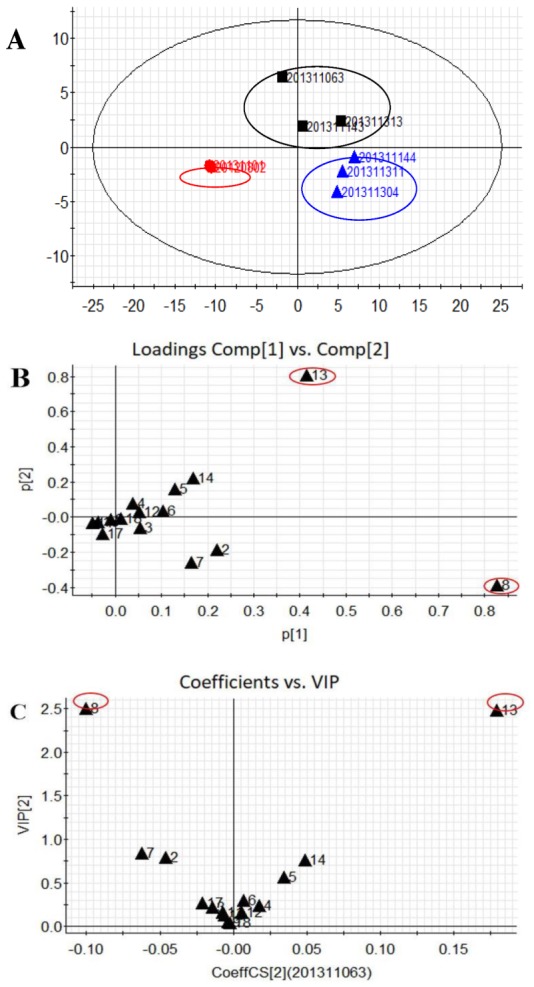
Principal component analysis (PCA) score plot (**A**), PCA loading plot (**B**), and VIP score plot from partial least square discriminant analysis (PLS-DA) (**C**).

Obvious classification was further performed through principal component analysis (PCA), shown in Figure 4. All the 8 HGT samples were readily classified into three groups based on their different manufacturers (Figure 4A). The corresponding PCA loading plot is illustrated in Figure 4B. Obviously, two compounds, isochlorogenic acid A (**8**) and saikosaponin A (**13**), were unambiguously identified as Q-markers for HGT samples. Furthermore, the VIP plot from partial least square discriminant analysis (PLS-DA) confirmed that these three characteristic Q-markers had larger VIP values of more than 2.5 than those of other compounds (Figure 4C). Therefore, it is unreasonable that only some simple lignans were chosen as index components for quality control of HGT.

## 3. Materials and Methods

### 3.1. Chemicals and Materials

HPLC-grade acetonitrile (ACE) was purchased from the Dikama Technology Corporation (Richmond Hill, NY, USA). Deionized water was prepared in a Milli-Q system (Millipore, Bedford, MA, USA) and used throughout the study. Standards of compounds **1**–**18** were purchased from the Chengdu JSMT Biotechnology Co., Ltd. (Chengdu, China). The purity of each standard compound was determined to be more than 98% by normalization of the peak areas detected by HPLC-ELSD analysis. All other reagents were of analytical grade.

### 3.2. Preparation of Standard Solutions

The standards for schisandrin C, schisandrol A, schisandrol B, schisantherin A, schisantherin B, schizandrin A, schizandrin B, isochlorogenic acid A, kaempferitrin, epigoitrin, scopoletin, saikosaponin C, saikosaponin A, saikosaponin B_2_, saikosaponian D, hyodeoxycholic acid, indigo, and schisanhenol were weighed accurately and dissolved in methanol at a concentration of 1 mg mL^−1^. A mixed intermediate stock standard solution was then prepared in methanol; the concentrations of compounds **1**–**18** in this solution were 40 ug.ml^−1^ In addition, the working standard solutions containing each of the target compounds were prepared by diluting the standard stock solutions with methanol to a series of appropriate concentrations. The standard stock solutions and the working standard solutions were stored at 4 °C.

### 3.3. Samples Preparation

Eight batches of HT were collected from different manufacturers. Commercial products **1**–**3** (lot no.201311063, 201311143, 201311144), **4**–**6** (lot no.1311311, 1311304, 1311313), and **7** and **8** (lot no.20131101, 20120802) were purchased from Kuihua Pharmaceutical Co., Ltd. (Heilongjiang, China), Shiyitang Pharmaceutical Co., Ltd. (Harbin, China), and Huayu Pharmaceutical Co., Ltd. (Harbin, China), respectively. All sample solutions were ultrasonically extracted with 20 mL methanol for 20 min at 40 °C and all samples were filtered through a 0.22 µm membrane filter.

### 3.4. Chromatographic and MS Conditions

Analysis was performed using an Acquity UPLC system with a conditioned autosampler at 4 °C. Chromatographic separation was carried out at 35 °C on an Acquity UPLC HSS T3 column (1.8 um, 2.1 mm × 100 mm). The mobile phase was composed of 0.1% formic acid in water (A) and 0.1% formic acid in acetonitrile (B) with a gradient elution: 0–4 min, 75–15% (A); 4–8 min, 15–12% (A); 8–10 min, 12–0% (A) and 10–12 min, 0–70% (A), until the end of the run at 12.0 min. The injection volume was 2 μL.

The mass spectrometry was performed on a 4000 QTRAP LC-MS/MS system (AB SCIEX) equipped with an ESI interface in negative mode. All instruments were controlled and synchronized by Analyst software (version 1.6, SCIEX). The ion spray voltage was set at 3300) V, turbo spray temperature was 550 °C, and the interface heater was on. Both nebulizer gas (gas 1) and heater gas (gas 2) were set at 55 psi.

## 4. Conclusions

The quality analysis of the multiple components in Chinese patent medicine prescriptions has met faced emerging challenges from the structural complexities in the co-occurrence of water-soluble and fat-soluble components in HGT. This was the first report of the simultaneous determination of the 18 major compounds in HGT using UHPLC-ESI-MS/MS method coupled with a MRM mode. In addition, these 18 compounds were unambiguously classified into seven structural types attributed to lignans, organic acids, flavonoids, alkaloids, coumarins, saponins, and bile acids. This novel evaluation approach was not only environmentally friendly but also a more efficient approach for quantitative analysis of HGT samples. This method provides an excellent example for quality assessments of Chinese patent medicine prescriptions due to its high capacity, high sensitivity, and high selectivity.

## Figures and Tables

**Table 1:** Values determined by the developed ultrahigh performance liquid chromatography–electrospray ionization multiple reaction monitoring tandem mass spectrometry (UHPLC-ESI-MRM-MS/MS) method.

No.	*t*_R_ (min)	Compounds	Molecular Weights	Precursor Ions (*m*/*z*)	Q_1_ (Da)	Q_3_ (Da)	DP	CE
1	5.77	Schisandrin C	384.42	[M + Na]^+^	406.8	161.0	98.79	47.07
2	5.99	Schisandrol A	432.51	[M + H − H_2_O]^+^	415.8	339.0	113.26	47.12
3	6.25	Schisandrol B	416.47	[M + H − H_2_O]^+^	399.9	300.1	100.74	47.95
4	7.00	Schisantherin A	536.57	[M + Na]^+^	559.9	341.2	160.59	45.66
5	7.09	Schisantherin B	514.57	[M + Na]^+^	537.7	341.1	159.39	39.61
6	8.30	Schizandrin A	416.51	[M + H]^+^	417.7	301.2	157.41	44.09
7	8.70	Schizandrin B	400.47	[M + H]^+^	401.7	285.1	149.92	36.85
8	1.52	Isochlorogenic acid A	354.31	[M − H]^−^	353.3	190.8	−63.68	−21.72
9	1.56	Kaempferitrin	578.57	[M − H]^−^	577.3	285.0	−131.67	−47.04
10	2.27	Epigoitrin	129.18	[M − H]^−^	127.9	58.0	−45.86	−15.63
11	3.12	Scopoletin	192.17	[M − H]^−^	190.9	103.7	−65.39	−35.86
12	4.34	Saikosaponin C	927.12	[M − H]^−^	926.0	617.2	−169.58	−51.86
13	4.96	Saikosaponin A	780.98	[M − H]^−^	779.4	617.7	−195.03	−48.56
14	5.16	Saikosaponin B_2_	780.99	[M − H]^−^	779.5	617.8	−184.29	−48.30
15	5.79	Saikosaponian D	780.99	[M − H]^−^	779.6	617.5	−185.68	−52.11
16	5.53	hyodeoxycholic acid	392.56	[M − H]^−^	391.7	374.3	−166.39	−45.04
17	6.14	Indigo	262.26	[M − H]^−^	261.1	155.8	−105.36	−44.00
18	7.36	Schisanhenol	402.5	[M − H]^−^	401.1	339.2	−89.91	−34.67

Q_1_: quadrupole one; Q_3_: quadrupole three; CE: collision energy.

**Table 2:** Summary of calibration results, limit of detection (LOD), and limit of quantification (LOQ) values.

No.	Regression Equation	Linea Range (μg/mL)	*R* ^2^	LODs (μg/mL)	LOQs (μg/mL)
1	*y* = 655.51*x* − 184.48	0.63–10.00	0.999	0.16	0.31
2	*y* = 219418*x* + 32705	0.08–5.00	0.998	2.44 × 10^−3^	9.77 × 10^−3^
3	*y* = 152722*x* + 2964	0.08–2.50	1.000	2.44 × 10^−3^	9.77 × 10^−3^
4	*y* = 71665*x* + 1342.6	0.01–0.63	0.999	9.77 × 10^−3^	0.02
5	*y* = 10172*x* + 60.62	0.08–2.50	0.999	9.77 × 10^−3^	0.02
6	*y* = 47841*x* + 2066.3	0.01–2.50	0.998	2.44 × 10^−3^	9.77 × 10^−3^
7	*y* = 16223*x* − 186.99	0.04–2.50	1.000	9.77 × 10^−3^	0.02
8	*y* = 31312*x* + 16578	0.01–5.00	0.999	2.44 × 10^−3^	9.77 × 10^−3^
9	*y* = 409356*x* − 550.49	0.002–0.08	1.000	2.44 × 10^−3^	9.77 × 10^−3^
10	*y* = 1123.6*x* − 1129.2	1.25–40.00	0.999	0.63	1.25
11	*y* = 70281*x* − 312.58	0.01–1.25	0.999	2.44 × 10^−3^	9.77 × 10^−3^
12	*y* = 100.12*x* + 64.52	0.63–40.00	0.999	0.04	1.25
13	*y* = 44268*x* + 30605	0.08–40.00	0.999	2.44 × 10^−3^	9.77 × 10^−3^
14	*y* = 156901*x* − 1354.7	0.01–0.63	0.999	2.44 × 10^−3^	9.77 × 10^−3^
15	*y* = 44456*x* + 71374	0.31–20.00	0.992	2.44 × 10^−3^	9.77 × 10^−3^
16	*y* = 147.67*x* + 569.89	0.63–40.00	0.991	0.04	0.16
17	*y* = 7702.2*x* − 121.23	0.04–1.25	0.999	0.02	0.04
18	*y* = 724.64*x* − 232.14	0.31–10.00	0.999	0.02	0.08

Note: *y* is the peak area of reference standards, and *x* is the value of the reference compound’s concentration (μg/mL).

**Table 3:** Contents (μg/g) of 18 marker compounds in 8 different batches of Hu Gan tablets.

Manufacturers	Batches	1	2	3	4	5	6	7	8	9	10	11	12	13	14	15	16	17	18
a1.	201311063	/	30.8	1.0	2.5	12.3	10.7	13.8	96.2	0.01	/	0.1	0.3	44.8	7.2	/	0.12	/	0.2
a2.	201311143	/	34.5	1.4	2.0	11.6	11.2	18.6	115.9	0.02	/	0.1	/	34.6	7.2	/	0.11	0.2	0.2
a3.	201311144	/	39.0	1.4	1.7	12.3	10.2	15.7	160.7	0.02	/	0.1	0.5	34.6	7.2	/	0.13	0.2	0.2
b1.	201311311	/	37.0	1.7	2.5	12.1	11.7	20.8	152.0	0.02	/	0.1	0.6	27.8	7.2	/	0.10	0.5	0.2
b2.	201311304	/	38.0	1.6	2.0	11.1	10.7	22.7	153.0	0.02	/	0.1	0.1	23.2	4.8	/	0.10	0.3	0.3
b3.	201311313	/	39.3	1.6	2.0	12.6	9.2	16.7	139.5	0.02	/	0.1	0.6	45.3	7.2	/	0.03	0.3	0.3
c1.	20131101	/	31.8	1.3	1.7	10.3	8.5	14.8	67.5	0.03	/	0.6	/	3.4	2.4	/	0.26	0.6	0.2
c2.	20120802	/	32.0	0.9	1.5	8.6	8.5	13.0	71.5	0.04	/	0.3	/	3.4	2.4	/	0.31	0.5	0.2

Note: a = Kuihua, Heilongjiang; b = Shiyitang, Harbin; c = Huayu, Harbin.

## Supplementary Materials

The Supplementary Materials are available online at https://www.mdpi.com/1420-3049/24/23/4241/s1.
