# Supplementary material for: UHPLC-ESI-MS/MS Quantitative Analyses of Multicomponent Hu Gan Tablets
Source: Molecules. 2019 Nov 21;24(23):4241. doi: 10.3390/molecules24234241 (PMC6930664; doi:10.3390/molecules24234241)
Supplement: Supplementary file 1 [file molecules-24-04241-s001.pdf]

Table S1. Intra- and inter-day variability for the assay of the 18 constituents

No.	Concentrations(μg/mL)	Intra-day			Inter-day		
		Found	RSD(%)	Accuracy(%)	Found	RSD(%)	Accuracy(%)
	10.00	9.67±0.13	1.34	96.71	10.15±0.08	0.79	101.54
**1**	5.00	5.08±0.06	1.18	101.63	5.10±0.11	2.16	102.08
	2.50	2.39±0.02	0.84	95.64	2.42±0.07	2.89	96.82
	10.00	10.32±0.03	0.29	103.24	11.00±0.06	0.55	110.03
**2**	5.00	5.22±0.10	1.92	104.46	4.93±0.14	2.84	98.66
	2.50	2.54±0.07	2.76	101.69	2.37±0.15	2.11	94.89
	10.00	10.03±0.05	0.50	100.37	9.75±0.10	1.03	97.57
**3**	5.00	5.13±0.03	0.58	102.65	5.09±0.11	2.16	101.85
	2.50	2.41±0.07	2.90	96.42	2.50±0.04	1.60	100.02
	10.00	9.58±0.18	1.88	95.80	9.66±0.07	0.72	96.60
**4**	5.00	4.89±0.06	1.23	97.81	4.93±0.10	2.03	98.61
	2.50	2.38±0.07	2.94	95.24	2.46±0.06	2.44	98.44
	10.00	10.12±0.12	1.19	101.25	9.84±0.19	1.93	98.45
**5**	5.00	4.81±0.14	2.91	96.22	5.18±0.03	0.58	103.62
	2.50	2.57±0.05	1.95	102.88	2.49±0.06	2.41	99.68
	10.00	9.89±0.08	0.81	98.94	9.92±0.05	0.50	99.24
**6**	5.00	5.06±0.15	2.96	101.29	5.07±0.13	2.56	101.49
	2.50	2.60±0.07	2.69	104.03	2.63±0.09	3.42	105.23
	10.00	9.75±0.08	0.82	97.53	10.14±0.11	1.08	101.43
**7**	5.00	5.17±0.13	2.51	103.46	5.17±0.15	2.90	103.46
	2.50	2.61±0.02	0.77	104.41	2.62±0.04	1.53	104.81
	10.00	10.23±0.18	1.76	102.34	10.25±0.05	0.49	102.54
**8**	5.00	4.83±0.04	0.83	96.69	5.00±0.00	0.00	100.00
	2.50	2.49±0.00	0.00	99.63	2.56±0.06	2.34	102.43
	10.00	10.17±0.06	0.59	101.76	10.20±0.17	1.67	102.06
**9**	5.00	4.96±0.11	2.22	99.21	4.99±0.12	2.40	99.81
	2.50	2.39±0.04	1.67	95.64	2.45±0.02	0.82	98.04
	10.00	9.87±0.05	0.51	98.74	10.10±0.08	0.79	101.04
**10**	5.00	5.16±0.04	0.78	103.28	5.18±0.01	0.19	103.68
	2.50	2.61±0.06	2.30	104.43	2.57±0.10	3.89	102.83
	10.00	10.21±0.11	1.08	102.15	10.19±0.10	0.98	101.95
**11**	5.00	5.13±0.13	2.53	102.67	5.16±0.14	2.71	103.27
	2.50	2.55±0.07	2.75	102.09	2.59±0.03	1.16	103.69
	10.00	9.87±0.04	0.41	98.76	9.91±0.09	0.91	99.16
**12**	5.00	5.05±0.08	1.58	101.07	5.12±0.15	2.93	102.47
	2.50	2.44±0. 05	2.05	97.66	2.46±0.06	2.44	98.46
	10.00	10.12±0.09	0.89	101.28	10.14±0.06	0.59	101.48

**13**	5.00	4.84±0.07	1.45	96.88	4.93±0.07	1.42	98.68
	2.50	2.58±0.04	1.55	103.29	2.58±0.01	0.39	103.29
	10.00	9.69±0.02	0.21	96.96	9.77±0.04	0.41	97.76
**14**	5.00	5.20±0.11	2.12	104.03	5.18±0.08	1.54	103.63
	2.50	2.48±0.05	2.02	99.20	2.51±0.07	2.79	100.40
	10.00	9.83±0.07	0.71	98.35	10.04±0.05	0.50	100.45
**15**	5.00	4.88±0.07	1.43	97.62	4.95±0.11	2.22	99.02
	2.50	2.54±0.06	2.36	101.66	2.60±0.04	1.54	104.06
	10.00	10.36±0.09	0.87	103.67	10.37±0.05	0.48	103.77
**16**	5.00	5.06±0.01	0.20	101.22	5.11±0.03	0.59	102.22
	2.50	2.39±0.03	1.26	95.65	2.42±0.06	2.48	96.85
	10.00	10.24±0.21	2.05	102.43	10.33±0.15	1.45	103.33
**17**	5.00	4.90±0.06	1.22	98.08	4.98±0.02	0.40	99.68
	2.50	2.57±0.10	3.89	102.84	2.59±0.09	3.47	103.64
	10.00	9.84±0.15	1.52	98.45	9.87±0.10	1.01	98.75
**18**	5.00	5.16±0.08	1.55	103.26	5.18±0.11	2.12	103.66
	2.50	2.65±0.04	1.51	106.01	2.65±0.02	0.75	106.00

Table S2 Recovery and matrix effect of reference standards

No.	Original (mg/g)	Spiked (mg)	Found (mg)	Mean recovery (%)	RSD (%) (n = 3)
		0.40	1.82	85.00	2.11
2	1.48	0.80	2.35	108.75	1.84
		1.50	3.03	103.33	0.09
		1.70	8.61	107.65	1.42
3	6.78	3.40	10.08	97.06	2.07
		6.80	13.66	101.18	3.00
		0.30	1.20	70.00	1.55
4	0.99	0.50	1.47	96.00	2.16
		1.00	1.87	88.00	0.08
		1.20	5.91	90.83	1.04
5	4.82	2.40	7.09	94.58	2.08
		4.80	9.51	97.71	1.49
		1.20	5.84	94.17	1.26
6	4.71	2.40	7.34	109.58	2.10
		4.70	9.25	96.60	1.07
		1.90	9.34	88.42	2.53
7	7.66	3.80	12.10	116.84	0.85
		7.70	14.96	94.81	1.71
		2.20	10.81	98.64	1.94
8	8.64	4.30	12.83	97.44	2.79
		8.60	17.19	99.42	2.66
		0.0005	0.0026	120.00	0.42
9	0.002	0.001	0.0029	90.00	0.81
		0.002	0.0038	90.00	1.26
		0.021	0.080	80.95	1.37
11	0.063	0.044	0.111	109.09	2.41
		0.065	0.132	106.15	2.30
		0.025	0.092	112.00	1.18
12	0.064	0.030	0.097	110.00	2.04
		0.069	0.134	101.45	1.26
		1.30	6.11	93.08	2.05
13	4.90	2.50	7.36	98.40	1.53
		4.90	9.74	98.78	0.69
		0.010	0.039	80.00	0.16
14	0.031	0.023	0.060	126.09	1.51
		0.036	0.069	105.56	0.22
		0.40	1.61	77.50	1.18
16	1.30	0.70	1.88	82.86	2.04
		1.30	2.62	101.54	1.65
		0.02	0.09	100.00	0.51
17	0.07	0.04	0.11	100.00	0.47
		0.07	0.13	85.71	1.17
		0.10	0.51	80.00	0.93
18	0.43	0.30	0.69	86.67	0.77
		0.50	0.89	92.00	1.32
